# Hemodynamic Characteristics of Mechanically Ventilated COVID-19 Patients: A Cohort Analysis

**DOI:** 10.1155/2021/8882753

**Published:** 2021-01-04

**Authors:** E. Christiaan Boerma, Carina Bethlehem, Franciena Stellingwerf, Fellery de Lange, Koen W. Streng, Peter M. Koetsier, Inge T. Bootsma

**Affiliations:** Department of Intensive Care, Medical Centre Leeuwarden, Leeuwarden, Netherlands

## Abstract

**Background:**

Solid data on cardiovascular derangements in critically ill COVID-19 patients remain scarce. The aim of this study is to describe hemodynamic characteristics in a cohort of COVID-19-related critically ill patients.

**Methods:**

A retrospective observational cohort study in twenty-eight consecutive mechanically ventilated COVID-19 patients. Pulse contour analysis-derived data were obtained from all patients, using the PiCCO® system.

**Results:**

The mean arterial pressure increased from 77 ± 10 mmHg on day 1 to 84 ± 9 mmHg on day 21 (*p*=0.04), in combination with the rapid tapering and cessation of norepinephrine and the gradual use of antihypertensive drugs in the vast majority of patients. The cardiac index increased significantly from 2.8 ± 0.7 L/min/m^2^ on day 1 to 4.0 ± 0.8 L/min/m^2^ on day 21 (*p* < 0.001). Dobutamine was administered in only two patients. Mean markers of left ventricular contractility and peripheral perfusion, as well as lactate levels, remained within the normal range. Despite a constant fluid balance, extravascular lung water index decreased significantly from 17 ± 7 mL/kg on day 1 to 11 ± 4 mL/kg on day 21 (*p* < 0.001). Simultaneously, intrapulmonary right-to-left shunt fraction (*Q*_s_/*Q*_t_) decreased significantly from 27 ± 10% in week 1 to 15 ± 9% in week 3 (*p*=0.007). PaO_2_/FiO_2_ ratio improved from 159 ± 53 mmHg to 319 ± 53 mmHg (*p* < 0.001), but static lung compliance remained unchanged.

**Conclusions:**

In general, this cohort of patients with COVID-19 respiratory failure showed a marked rise in blood pressure over time, not accompanied by distinctive markers of circulatory failure. Characteristically, increased extravascular lung water, vascular permeability, and intrapulmonary shunt diminished over time, concomitant with an improvement in gas exchange.

## 1. Introduction

By the spring of 2020, the impact of the COVID-19 pandemic started to materialize. All over the globe, the intensive care unit (ICU) community is faced with overwhelming amounts of patients in need of ICU treatment, predominantly due to acute respiratory failure [1–3]. Efforts are made to understand the rather unique phenotype of a lung disease that fulfills the Berlin criteria for acute respiratory distress syndrome [4, 5]. It is conceivable that the profound hypoxia in COVID-19 patients is not only a consequence of impaired ventilation, or high incidence of pulmonary embolism, but may also be related to circulatory failure in general [6]. Furthermore, COVID-19 infection is not limited to the lungs but involves other organs as well, including the renal and cardiovascular system [7, 8]. Several mechanisms related to organ dysfunction beyond the primary target organ have been suggested, including circulatory failure as a result of hyperinflammation, microthrombosis, and heart failure [9, 10]. However, solid data on cardiovascular derangements in critically ill COVID-19 patients remain scarce. The aim of this study is to describe hemodynamic characteristics in a cohort of COVID-19-related critically ill patients.

## 2. Methods

### 2.1. Setting and Patient Selection

This retrospective single-center study was performed in a closed-format 29-bed mixed ICU in a tertiary teaching hospital. All consecutive patients ≥18 years admitted to the ICU during the period March 15 to April 30, 2020, with PCR-confirmed COVID-19 and the need for invasive mechanical ventilation were included in the study. All patients were included in the study within the first 2 hours after intubation. There were no exclusion criteria. The study was performed in accordance with the Declaration of Helsinki, and anonymized data were used for analysis. According to applicable laws, the need for individual consent was waived by a local ethics committee (RTPO nWMO 2020 0027, Regionale Toetsingscommissie Patientgebonden Onderzoek, Leeuwarden, the Netherlands).

### 2.2. Protocol

A dedicated COVID-19 protocol concerning diagnostics and treatment was designed and approved by the local ICU team before admission of the first COVID-19 patient. In case of respiratory failure, ventilatory support was restricted to invasive mechanical ventilation. Directly after the initiation of mechanical ventilation, a central venous line in the internal jugular vein was inserted, as well as an arterial line in the femoral or brachial artery. Pulse contour analysis-derived data were obtained by a PiCCO® system (Getinge AB, Gothenburg, Sweden). A minimal mean arterial pressure (MAP) was set at 65 mmHg, and norepinephrine (NE) was the only available vasopressor. A restrictive policy with respect to fluid administration was maintained throughout the entire ICU admission. In the first week of ICU admission, the preferred ventilatory setting was pressure-regulated volume control (PRVC) in combination with midazolam/fentanyl sedation to a Richmond Agitation-Sedation Scale (RASS) level of −4 to −5 and additional muscle paralysis with rocuronium bromide if deemed necessary [11]. Lung-protective mechanical ventilation was applied, using tidal volumes of 6 ml/kg predicted body weight, positive end-expiratory pressure (PEEP) levels between 8 and 12 cm H_2_O, and driving pressure <15 cm H_2_O. High fractions of O_2_ as well as mild hypercapnia (until pH 7.20–7.25) were accepted. Thrombosis prophylaxis was unaltered during the pandemic and restricted to a normal dose of low molecular weight heparin (nadroparine 0.3 ml subcutaneously, once a day).

### 2.3. Data Collection

The following data were extracted from the patient data management system (Epic®, Verona, Wisconsin, USA): at baseline: demographic characteristics, comorbidities (diabetes, hypertension, and immune-compromised status), and medication (angiotensin-converting enzyme (ACE) inhibitor or angiotensin II receptor blocker). Severity of illness scores (Acute Physiology and Chronic Health Evaluation (APACHE) III and Sequential Organ Failure Assessment (SOFA)) were calculated over the first 24 hours following ICU admission [12, 13]. Furthermore, the maximum SOFA score, need for renal replacement therapy, prone positioning, and number of days in ICU were documented. On a daily basis, fluid balance and body weight were recorded. Delirium was defined as the presence of one or more days with a positive Confusion Assessment Method for the ICU (CAM-ICU) score [14]. Pulmonary embolism was confirmed by CT-scan; aspergillosis was diagnosed by a combination of CT-imaging and a positive galactomannan antigen titer in a bronchoalveolar lavage.

Data were collected for a maximum of 21 consecutive days, or until ICU discharge or death before day 21. Day 21 was chosen since this was the time by which 90% of all patients were no longer ventilated. The following (semicontinuous) hemodynamic data were extracted once daily at 23.59: MAP, heart rate (HR), central venous pressure (CVP), perfusion index (PI; IntelliVue®, Philips Medical Systems, Best, the Netherlands), cardiac index (CI), stroke volume index (SV_i_), and left ventricular contractility index (dPmax). Global end-diastolic volume index (GEDV_i_), extravascular lung water index (EVLW_i_), and pulmonary vascular permeability index (PVP_i_) were recorded during daily calibration of the PiCCO® system.

Ventilation parameters were recorded once daily simultaneously with hemodynamic data: mode of mechanical ventilation, PEEP settings, fraction of inspired oxygen (FiO_2_), plateau pressure (P_plat_), static compliance (C_stat_), and PaO_2_/FiO_2_ (P/F) ratio. Intrapulmonary right-to-left shunt fractions (*Q*_s_/*Q*_t_) were calculated according to Gattinoni et al. and collected once a week [15]. Daily laboratory parameters included blood gas analysis, lactate, creatinine, and sodium. Troponin-T, D-dimer, and procalcitonin were measured twice weekly.

### 2.4. Statistical Analysis

The data are expressed as mean ± standard deviation (SD). The Statistical Package for Social Sciences (SPSS 24 for Windows, Chicago, IL, USA) was used for descriptive statistical analysis. Comparison of data was restricted to the difference between day 1 and day 21. Statistical analysis was performed with applicable nonparametric tests for paired data (Wilcoxon), due to the limited number of patients in this study. A *p* value <0.05 was considered statistically significant. Correction for multiple comparisons was not performed.

## 3. Results

### 3.1. General

During the six-week inclusion period, 30 consecutive patients with polymerase chain reaction-confirmed COVID-19 were admitted to our ICU. Two patients did not need mechanical ventilation and were excluded from further analysis. The mean age was 67 ± 9 years, and additional baseline characteristics are summarized in Table 1. Twelve patients (43%) were admitted after transfer from a primary ICU for logistical reasons, with a mean prior stay of 3 ± 2 days. Delirium was the most common complication (95%).

### 3.2. Hemodynamic Data

**Figure 1:**
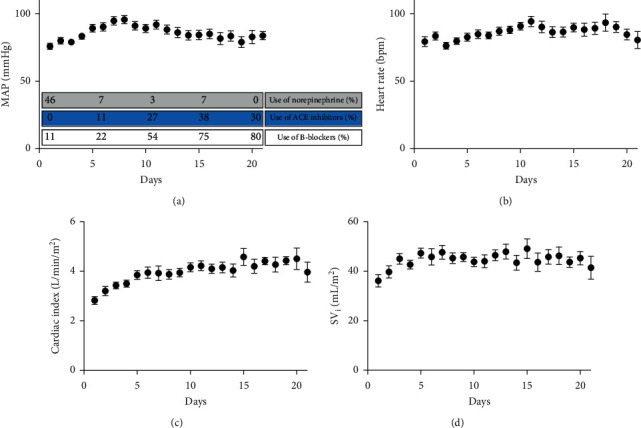
Circulatory characteristics (I) of COVID-19 patients. MAP: mean arterial pressure; bpm: beats per minute; SV_i_: stroke volume index.

The overall blood pressure was well-preserved. On day 1, the mean MAP was 77 ± 10 mmHg; 46% of patients needed vasopressive support with a mean NE dose of 0.04 ± 0.09 *µ*g/kg/min (Figure 1). Over time, the MAP increased significantly to 84 ± 9 mmHg on day 21 (*p*=0.04) in combination with a swift and complete weaning from NE administration in all patients. Simultaneously, a prompt and gradual increase in the administration of (a combination of) antihypertensive drugs was observed, including ACE inhibitors, *β*-blockers, and calcium antagonists (Figure 1).

The heart rate remained stable over time, from 80 ± 19 bpm on day 1 to 81 ± 19 bpm on day 21. In this period, the percentage of patients on *β*-blockers increased from 11% to 80% (Figure 1).

**Figure 2:**
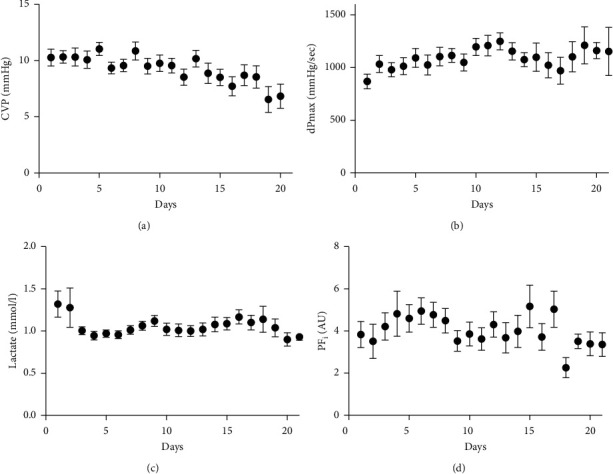
Circulatory characteristics (II) of COVID-19 patients. CVP: central venous pressure; PF_i_: peripheral perfusion index.

The cardiac index increased significantly over time, from 2.8 ± 0.7 L/min/m^2^ on day 1 to 4.0 ± 0.8 L/min/m^2^ on day 21 (*p* < 0.001; Figure 1). In only two patients, dobutamine was administered during less than 48 hours. In addition, CVP decreased gradually from 10 ± 4 mmHg on day 1 to 6 ± 3 mmHg on day 20 (Figure 1). CVP is represented in Figure 2. Throughout the study period, serum mean lactate values and the perfusion index remained within the normal range (Figure 2).

**Figure 3:**
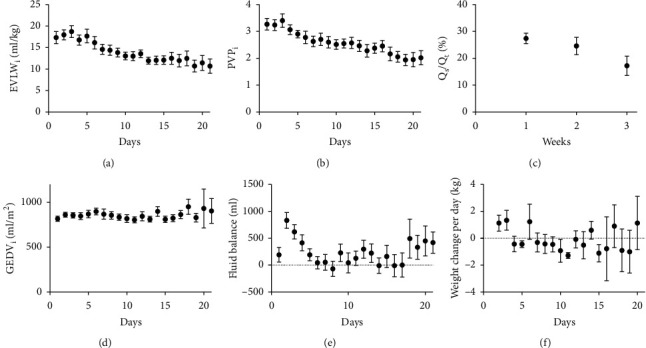
Circulatory characteristics (III) of COVID-19 patients. EVLW_i_: extravascular lung water index; PVP_i_: pulmonary vascular permeability index; *Q*_*s*_*/Q*_*t*_: pulmonary shunt fraction; GEDV_i_: global end-diastolic volume index.

Despite a constant GEDV_i_ and fluid balance throughout the study period, EVLW_i_ decreased significantly from 17 ± 7 mL/kg on day 1 to 11 ± 4 mL/kg on day 21 mL/kg (*p* < 0.001), in combination with a significant decrease in PVP_i_ (3.3 ± 1.1 and 2.0 ± 0.6 respectively, *p* < 0.001) (Figure 3). Simultaneously, *Q*_s_/*Q*_t_ decreased significantly from 27 ± 10% in week 1 to 15 ± 9% in week 3 (*p*=0.007; Figure 3).

### 3.3. Ventilation Data

**Figure 4:**
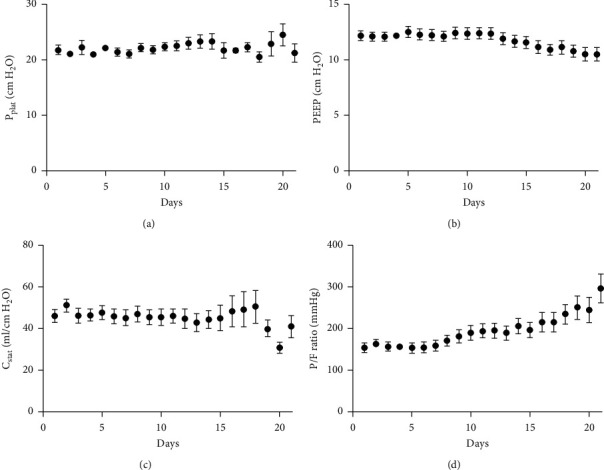
Ventilatory characteristics of COVID-19 patients. P_plat_: plateau pressure; PEEP: positive end-expiratory pressure; C_stat_: static compliance; P/F ratio: PaO_2_/FiO_2_ ratio.

The mean duration of mechanical ventilation was 17 ± 4 days; the mean number of days of muscle relaxants administration was 11 ± 5. During mechanical ventilation, P_plat_ and C_stat_ remained within close range (Figure 4). PEEP was gradually but modestly tapered over time from 10 ± 2 cm H_2_O on day 1 to 8 ± 2 cm H_2_O on day 21 (Figure 4). In the same period, P/F ratio improved significantly from 159 ± 53 mmHg to 319 ± 53 mmHg, *p* < 0.001 (Figure 4). Mean pH and CO_2_ remained stable over time (pH 7.38 ± 0.06 on day 1 to 7.26 ± 0.62 on day 21, *p*=0.07 and CO_2_ 5.8 ± 1.1 kPa on day 1 to 5.4 ± 1.7 kPa on day 21, *p*=0.44, respectively).

### 3.4. Additional Data

Additional data are presented in Supplementary Materials. Of note, the steep rise and gradual decline of D-dimer, as well as the increase of Troponin-T over time, has been depicted (Supplementary Figures 1 and 2).

## 4. Discussion

From these observational data, a consistent picture emerges. In the vast majority of patients, blood pressure is well maintained above 65 mmHg with a very modest dose of NE in only half of the patients at the start of mechanical ventilation. Over time, there is a striking tendency to hypertension in almost all patients, with a subsequent need for (a combination of) antihypertensive drugs, despite the substantial use of sedatives. In addition, signs of circulatory failure were infrequent. Markers of macrohemodynamic blood flow (CI), left ventricular contractility (dPmax), and preload (GEDV_i_) were generally well within the normal range, without substantial administration of fluids or inotropic drug support. In addition, mean values of lactate levels, as a surrogate marker for organ perfusion, and PF index, as an indicator of peripheral perfusion, were not outside reference values. The improvement over time in gas exchange (P/F ratio) is accompanied by a marked decrease in markers of permeability-related lung oedema (EVLW_i_/PVP_i_) and intrapulmonary right-to-left shunt (*Q*_s_/*Q*_t_).

Our data are in line with previous observations of an increased *Q*_s_/*Q*_t_ as a characteristic feature of COVID-19 lung disease, combined with a relatively mild reduction in compliance, as compared with the more classical ARDS [4, 5]. Data on cardiac output are very scarce, but our observations seem in line with the reported baseline cardiac output in a small intervention study during supine positioning in COVID-19 lung disease [16]. Although clearly elevated, the observed shunt fractions in our study are somewhat lower than previously reported [17]. The characteristic tendency to the development of hypertension is in line with the suggested interference of the coronavirus with the renin-angiotensin-aldosterone system [18]. The well-described specific binding of COVID-19 to the ACE-II receptor has been associated with a rise in plasma angiotensin II levels and was linked to the viral load and lung injury [19]. The increase in concentration of an endogenous vasopressor apparently counteracted the histamine-induced vasodilation associated with long-term use of non-depolarizing neuromuscular blockage agents [20]. However, an additional effect of the observed mild hypercarbia cannot be ruled out.

In general, cardiac performance was well-preserved in our patients, despite the observed rise in troponin-T, previously postulated to be a direct marker of myocardial injury in COVID-19 disease [9, 21].

Due to the retrospective single-center design of this study, the interpretation of the data does not allow for firm general conclusions, including cause-and-effect relationships. The moderately high severity of illness scores may suggest a form of patient selection, due to local settings. Alternatively, when COVID itself has a moderate impact on systemic hemodynamic variables, this will be reflected by a limited rise in severity of illness scores too. We believe that the specific setting, including a well-protocolized treatment and standardized invasive hemodynamic monitoring in a homogeneous population with confirmed COVID-19 infection and limited comorbidities, offers a unique possibility to increase our knowledge on the hemodynamic features of COVID-19 disease. Due to the descriptive nature of this study, a preset primary endpoint was not formulated. Under these conditions, calculation of statistical differences has limited value but guides the reader to some extent in the interpretation of the graphically presented data.

## 5. Conclusion

In conclusion, our observational hemodynamic data in a cohort of mechanically ventilated patients with COVID-19 respiratory failure show a marked rise in blood pressure over time, not accompanied by distinctive markers of circulatory failure. Characteristically, increased extravascular lung water, vascular permeability, and intrapulmonary shunt diminished over time, concomitant with an improvement in gas exchange.

## Figures and Tables

**Table 1:** Baseline and ICU characteristics.

Baseline	*n* = 28
Age, years	67 ± 9
Male, *n* (%)	20 (71)
BMI, kg/m^2^	30 ± 5
APACHE III score	70 ± 27
Predicted mortality APACHE IV, %	36 ± 24
SOFA score (day 1)	6 ± 3
SOFA score (max)	8 ± 2
Comorbidities, *n* (%)	
Diabetes	4 (14)
Hypertension	13 (46)
Immunocompromised	4 (14)
Medication, *n* (%)	
ACE inhibitor	5 (18)
Angiotensin II receptor blocker	1 (4)
Creatinine (*µ*mol/L)	93 ± 66

ICU period	

ICU interventions	
Mechanical ventilation, *n* (%)	28 (100)
Mechanical ventilation, days	17 ± 4
Prone positioning, *n* (%)	16 (57)
Muscle relaxants, *n* (%)	26 (93)
Muscle relaxants, days	11 ± 5
Renal replacement therapy, *n*(%)	3 (11)
Complications, *n* (%)	
Pulmonary embolism	5 (18)
Aspergillosis	3 (11)
Delirium (CAM-ICU confirmed)	27 (95)
ICU survival, *n* (%)	21 (75)

The data are presented as mean ± SD, unless stated otherwise. BMI: body mass index; APACHE: Acute Physiology and Chronic Health Evaluation; SOFA: Sequential Organ Failure Assessment; ACE: angiotensin-converting enzyme; CAM-ICU: Confusion Assessment Method for the ICU [14].

## Data Availability

The datasets used and/or analyzed during the current study are available from the corresponding author on reasonable request. All data generated or analyzed during this study are included in this article and its supplementary information files.

## Abbreviations

ICU: Intensive care unit
MAP: Mean arterial pressure
NE: Norepinephrine
PRVC: Pressure-regulated volume control
RASS: Richmond Agitation-Sedation Scale
PEEP: Positive end-expiratory pressure
ACE: Angiotensin-converting enzyme
APACHE: Acute Physiology and Chronic Health Evaluation
HR: Heart rate
CVP: Central venous pressure
PI: Perfusion index
CI: Cardiac index
SV_i_: Stroke volume index
dPmax: Left ventricular contractility index
GEDV_i_: Global end-diastolic volume index
EVLW_i_: Extravascular lung water index
PVP_i_: Pulmonary vascular permeability index
PEEP: Positive end-expiratory pressure
FiO_2_: Fraction of inspired oxygen
P_plat_: Plateau pressure
C_stat_: Static compliance
P/F ratio: PaO_2_/FiO_2_ ratio
Q_*s*_/*Q*_t_: Intrapulmonary right-to-left shunt fractions.

## Supplementary Materials

Supplementary Materials Figure 1: coagulation and inflammatory parameters. Figure 2: serum sodium and creatinine.
