# Supplementary figures and images for: Hemodynamic Characteristics of Mechanically Ventilated COVID-19 Patients: A Cohort Analysis
Source: Crit Care Res Pract. 2021 Jan 4;2021:8882753. doi: 10.1155/2021/8882753 (PMC7783514; doi:10.1155/2021/8882753)

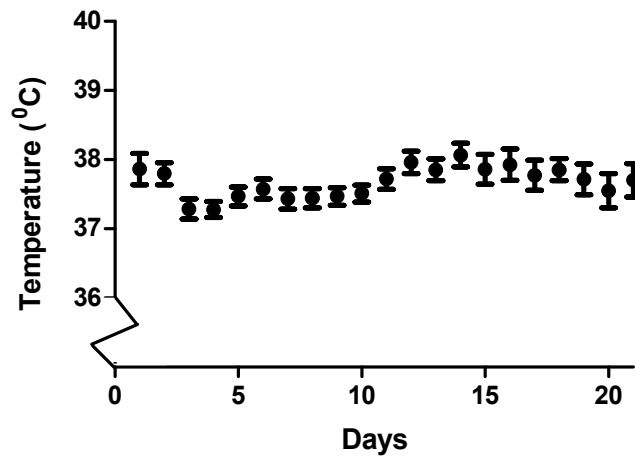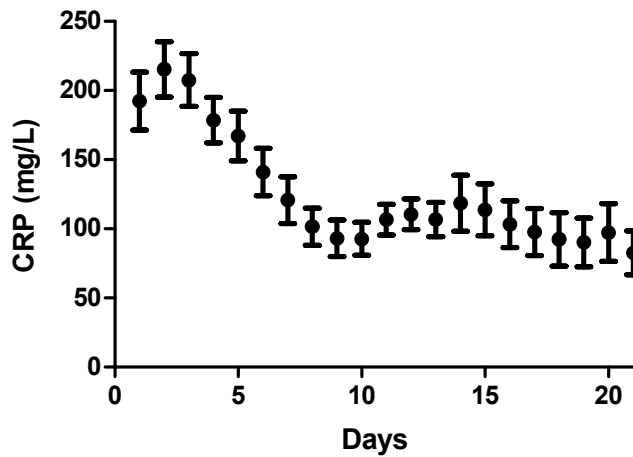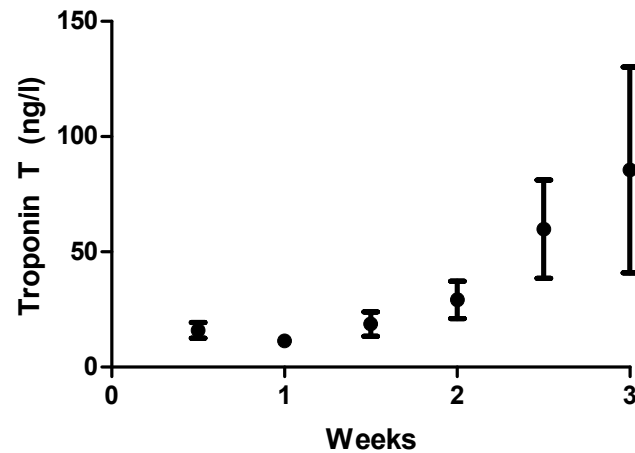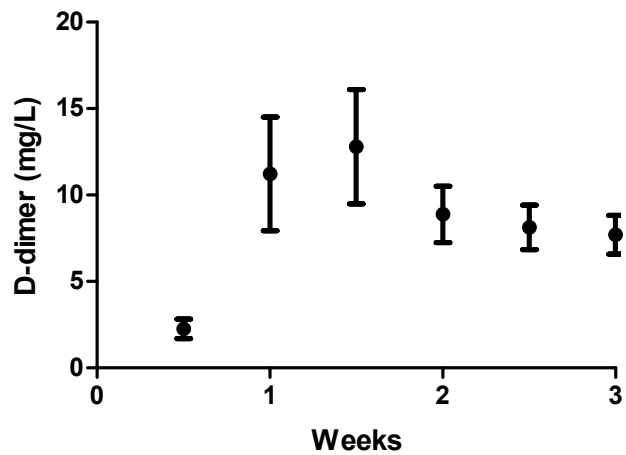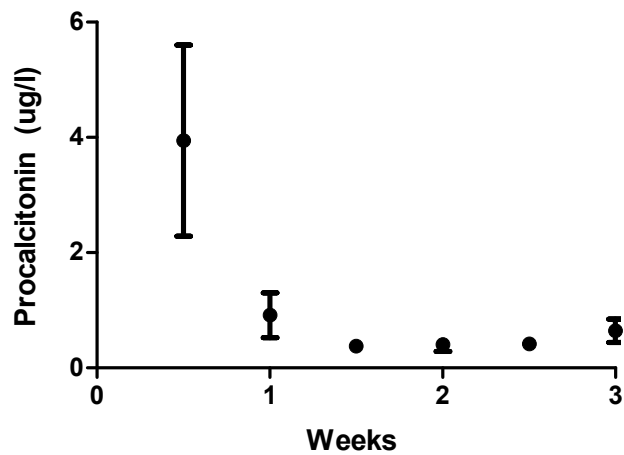

Supplement: Supplementary Materials — Figure 1: coagulation and inflammatory parameters. Figure 2: serum sodium and creatinine. [file 8882753.f1.zip › 8882753.f1/Supplemental Fig 1.pdf]

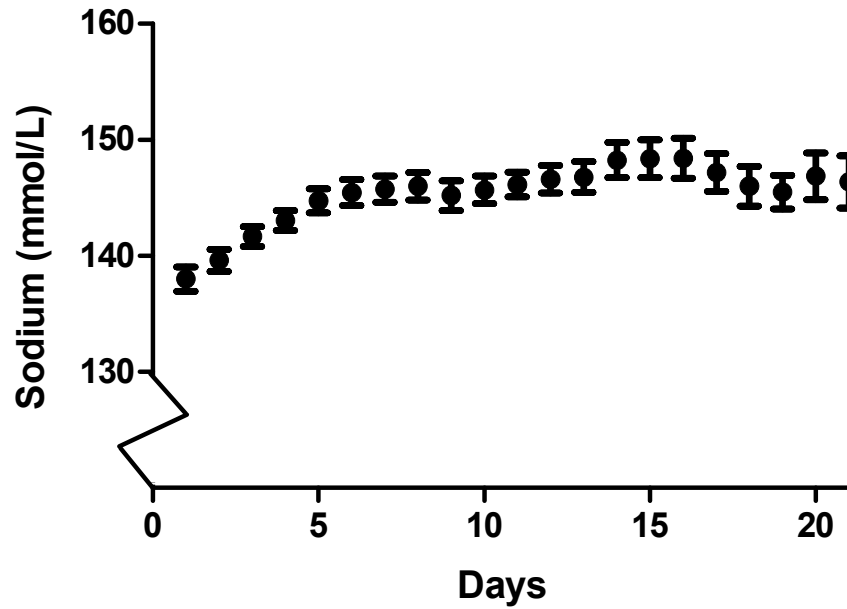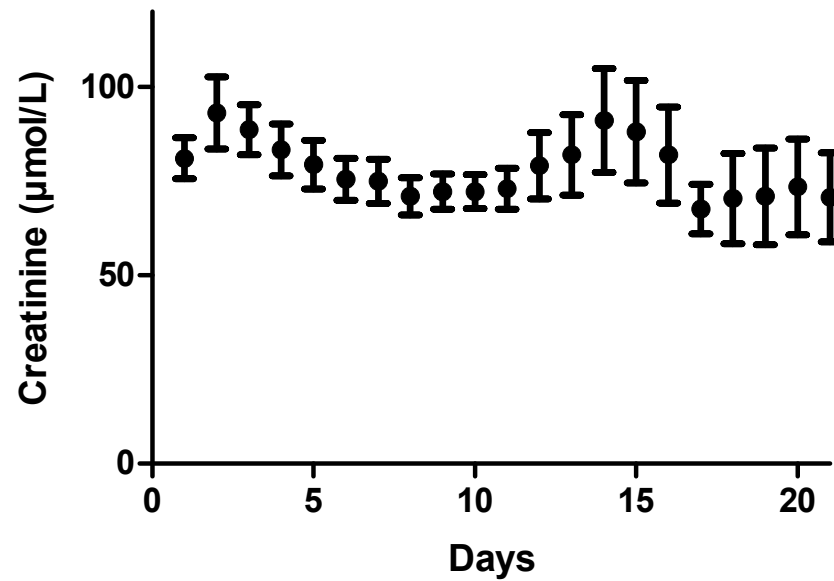

Supplement: Supplementary Materials — Figure 1: coagulation and inflammatory parameters. Figure 2: serum sodium and creatinine. [file 8882753.f1.zip › 8882753.f1/Supplemental Fig 2.pdf]
